# Laypeople’s Affective Images of Energy Transition Pathways

**DOI:** 10.3389/fpsyg.2018.01904

**Published:** 2018-10-10

**Authors:** Gisela Böhm, Rouven Doran, Hans-Rüdiger Pfister

**Affiliations:** ^1^Department of Psychosocial Science, Faculty of Psychology, University of Bergen, Bergen, Norway; ^2^Department of Psychology, Inland Norway University of Applied Sciences, Lillehammer, Norway; ^3^Institute of Experimental Industrial Psychology (LueneLab), Leuphana University of Lüneburg, Lüneburg, Germany

**Keywords:** energy transition, climate change, mental representation, affective imaging, free associations, Norway, Germany

## Abstract

This paper explores the public perception of energy transition pathways, that is, individual behaviors, political strategies, and technologies that aim to foster a shift toward a low-carbon and sustainable society. We employed affective image analysis, a structured method based on free associations to explore positive and negative connotations and affective meanings. Affective image analysis allows to tap into affective meanings and to compare these meanings across individuals, groups, and cultures. Data were collected among university students in Norway (*n* = 106) and Germany (*n* = 125). A total of 25 energy transition pathway components were presented to the participants who generated one free association to each component by indicating the first that came to mind when thinking of the component. Participants evaluated their associations by indicating whether they considered each association to be positive, negative, or neutral. These associations were coded by two research assistants, which resulted in 2650 coded responses in the Norwegian sample and 2846 coded responses in the German sample. Results for the two samples are remarkably similar. The most frequent type of association is a general evaluation of the component, for example concerning its valence or its importance. The second most frequent types of association are requirements needed to implement the component (e.g., national policies) and consequences of the component (e.g., personal or environmental consequences). Individual behaviors (e.g., walking) elicited thoughts about consequences and requirements, but also about the prevalence of such behaviors. Associations in response to technologies (e.g., carbon capture and storage) mainly referred to some descriptive aspect of the technology. Evaluations of the free responses were predominantly positive, but some components also elicited negative associations, especially nuclear power. The free associations that people generate suggest that they have vague and unspecific knowledge about energy transition pathways, that they process them in an automatic and intuitive rather than deliberative manner, and that they have clear affective evaluations of the presented components.

## Introduction

Energy transition commonly refers to “a change in the state of an energy system as opposed to a change in an individual energy technology or fuel source” (Grubler et al., 2016, p. 18). This may involve a heterogeneous multitude of potential changes including international agreements, national policies and regulations, industrial production and technological development, as well as changes to individual lifestyles, amongst others. Although the successful transition toward low-carbon and sustainable societies requires concerted changes at different levels, many aspects of this transition imply some sort of involvement of the public. Examples include public acceptance of policies, regulations, and technologies, as well as voting decisions. Public engagement with carbon reduction depends in part on the meanings that people ascribe to energy in everyday life (Whitmarsh et al., 2011).

This paper investigates the public perception of energy transition pathways, with a focus on subjective mental representations in the form of connotative meanings and affective images. These meanings and images have been shown to play an important part in shaping public perceptions and responses to societal risk issues such as climate change (Leiserowitz and Smith, 2017). The next section introduces the core concepts addressed throughout this paper covering energy transition pathways, mental models, and affective images. We will then present an exploratory study that investigated public perceptions of energy transition by eliciting free associations to various key terms related to the subject obtained from university students in two countries. Results of the study indicate which connotative meanings and affective images laypeople in these two samples ascribe to energy transition. Conclusions are drawn concerning the public understanding of this topic and likely implications for policy support and behavioral change toward a low-carbon society.

## Theoretical Background

### Energy Transition Pathways

Energy transition is a multifaceted concept that involves a variety of dimensions (Böhm et al., in press). One apparent dimension concerns the level of social aggregation at which a change takes place, ranging from individuals (e.g., energy saving at home or at work, purchasing of energy efficient appliances, avoiding car rides) to local and national governments (e.g., regulations such as green taxes) and international bodies (e.g., Paris agreement). Another possible dimension touches upon the distinction between supply-oriented (changes in energy production, e.g., renewable energy sources) and demand-oriented (changes in energy consumption, e.g., transportation modes) measures to transform energy systems. These various dimensions and levels rarely work in isolation, as can be illustrated by looking at renewable energies. Not only are energy sources from wind and solar embedded in a societal context of infrastructures and policies, but this context also has an impact on public support (Perlaviciute and Steg, 2014).

A large body of research exists in environmental psychology on topics that have some relevance to energy, yet little research has taken a comprehensive look at the many facets of energy transition. One existing research field deals with specific individual energy sources, for example perception and acceptance of nuclear power (for reviews, see Perlaviciute and Steg, 2014; Steg et al., 2015). Another existing research field tries to explain specific behaviors of individuals such as energy saving (e.g., turning down the heating, turning off the lights) or reduction in car driving (e.g., using public transport, car sharing) (for a review, see Steg and Vlek, 2009). In sum, these fields tend to focus on specific, often isolated, aspects of energy transition.

We argue that if the aim is to study subjective mental representations of energy transition, a broad range of potential actions and changes need to be taken into account (see also Böhm et al., in press). Henceforth, we will use the term *energy transition pathway* if we mean a combination of steps that are taken with the aim of reducing carbon emissions and improving the sustainability of energy use and production. An individual step such as a specific behavior, policy, or technology will be referred to as an *energy transition pathway component*.

### Mental Models

It is widely agreed that people’s subjective mental representations, or mental models, shape risk perceptions and play an important role in guiding behavior (Böhm and Pfister, 2001; Bostrom, 2017). Mental models comprise people’s knowledge about, and associations with, a phenomenon including causal inferences. They provide the basis for understanding a given event or situation; they also allow people to mentally simulate the future and to infer what will happen next (Bostrom, 2017). Mental models about a situation may include behavioral options, potential consequences of different behaviors, relevant actors and events, but also associations such as mental images, sounds, and smells. They can furthermore vary with respect to elaboration and range from full-fledged and detailed to vague and fragmentary representations (Böhm and Pfister, 2001).

An expanding literature suggests that mental models can guide individual behavior and policy support, for instance in response to climate change (e.g., Böhm and Pfister, 2001, 2017; Bostrom et al., 2012). Bostrom et al. (2012) demonstrated that people supported different climate policies depending on which factors they saw as the main causes of climate change. Engineering technologies were most supported by people who held a mechanistic model and considered natural events (e.g., volcano eruptions) the main cause of climate change. People who attributed climate change to carbon dioxide supported policies that specifically target carbon emissions (e.g., taxes on fossil fuels, carbon market), people with more vague conceptions about climate change supported unspecific green policies (e.g., funding research), and generally, people tended to support those policies they considered most effective in tackling the problem. Whilst several other studies have investigated mental models about climate change (e.g., Reynolds et al., 2010; for overviews, see Böhm and Pfister, 2001; Böhm, 2008; Bostrom, 2017), less is known about laypeople’s mental models about energy systems and their perceptions of different pathways to energy transition specifically. For example, which strategies does the public consider in the transition toward a low-carbon energy system, and what consequences do they anticipate, for example, for the economy, the environment, or society?

Mental models are not always detailed and elaborate, which may depend on how much a person knows and has thought about the issue in question. For global problems such as climate change, it has been found that people generally lack detailed conceptual understandings of the phenomenon (Leiserowitz and Smith, 2017). Given that energy transition is closely related to climate change (and that it is a new, similarly global and complex issue), people’s understanding of energy transition may also not be very elaborate. For this exploratory study, we therefore concluded that the public perception of energy transition may be better studied on the level of mental images rather than as detailed mental models with explicit, for example causal, judgments. One way of tapping into mental images is the elicitation of free associations, which allows studying the content of people’s minds without forcing them to express their thoughts in full language (cf. Leiserowitz and Smith, 2017).

Smith and Joffe (2013) analyzed free associations to climate change (they use the term global warming) from a social representations theory perspective, a theoretical approach that can explain the origin of mental images. These authors showed how strongly the socio-cultural context shapes people’s representations of global risk issues such as climate change. They argue that when trying to build a mental representation of an unfamiliar issue, people assimilate the new information to familiar structures, using symbols, icons, and metaphors that circulate in their socio-cultural context. In this respect, visual images are particularly important elements of the socio-cultural context because of their concreteness, ability to convey emotions and their status as expressive carriers of meaning in common sense thinking.

The assumption is that new information is encoded based on some familiar concept (‘anchoring’, Smith and Joffe, 2013). For climate change, it has been shown that laypeople often understand this phenomenon on the basis of their pre-existing mental models about ozone depletion, air pollution, or weather (Bostrom, 2017). A concrete representation of a new phenomenon is then created by transforming the familiar model through incorporating tangible images, concepts, and symbols (‘objectification,’ Smith and Joffe, 2013). For climate change, these may include images of melting ice, polar bears, flooding, smoking chimneys, or car exhaust pipes (see below). Smith and Joffe (2013) found that social representations of climate change were structured according to antinomic dyads, namely, self versus other, natural versus unnatural, and certainty versus uncertainty.

Smith and Joffe (2013) point out that the free associations revealed as part of their study mirrored the images that the British press used to depict climate change visually. Climate change, and presumably energy transition, are socially mediated phenomena in the sense that few aspects of it are personally experienced (Weber and Stern, 2011). An interesting question is thus whether traditional versus social media and new technologies assume different roles in shaping subjective mental representations. Comparisons of the themes and frames that are used in the coverage of the COP 21 summit in Paris (Painter et al., 2018) or coverage of the IPCC Fifth Assessment report (O’Neill et al., 2015) show that traditional print and online and social media are more similar than one might expect. An analysis of climate change debates on Twitter (Williams et al., 2015) showed that these discourses take place in part in homogenous attitudinal echo chambers constituting segregated and polarized camps of activists and skeptics, but also in mixed-attitude communities in which activists and skeptics interact. Exchanges between like-minded individuals tend to carry positive sentiment whereas messages in mixed-attitude communities are likely to express negative sentiment. These results indicate that social media have a strong potential to shape people’s associative mental images and to portray affect-laden meanings; as opposed to other forms of new technologies that seem likely to help build up an elaborate cognitively focused mental model (e.g., smart meter web portals, Mack and Tampe-Mai, 2016).

### Affective Images

It is increasingly recognized that information processing, risk perception, and decision-making are influenced by affect and emotions (Pfister and Böhm, 2008; Lerner et al., 2015). Dual systems theories see affect as being based on fast and intuitive, as opposed to analytic, processing (Epstein, 1994). Affective reactions often occur instantaneously and automatically and thus allow individuals to respond rapidly to their surroundings (Zajonc, 1980; Pfister and Böhm, 2008). Affect generally refers to an overall good or bad, positive or negative evaluation of an object, event, situation, idea, person, or other entity (e.g., Slovic et al., 2004). Leiserowitz and others (Leiserowitz, 2006; Lorenzoni et al., 2006; Leiserowitz and Smith, 2017) introduced the term ‘affective images’ to refer to mental images to which affective evaluations have become attached. Mental images include both perceptual and symbolic representations, that is, the whole range of sights, sounds, smells, ideas, words, symbols, or numbers. Affective images are all sorts of representations that carry affective meaning, with affective states becoming attached to mental images by learning and experience.

When it comes to research exploring public views on climate change, affective images have been linked with both risk perceptions and policy preferences. Smith and Leiserowitz (2012) tracked affective image associations to the term global warming among the American public over time in the period from 2002 to 2010. They identified significant trends; for example, ‘naysayer’ but also disaster images increased, while ice-melting images decreased. There was an overall trend to evaluate these associations negatively, with ‘naysayer’ images being among the strongest predictors of risk perception. These results document that affective images not only form a core element of people’s mental representations related to climate change but also shape people’s evaluations and, ultimately, their behaviors and policy preferences. Other research has shown that affect seems to be an integral part of social representations of climate change and energy-related topics (Fischer et al., 2012).

Truelove (2012) proposes a dual-process model of energy support, in which affective images and evaluations interact with cognitive evaluations in determining support for energy sources. Truelove’s study reports affective images for four energy sources: coal, nuclear power, natural gas, and wind. It also documented how each energy source is associated with specific mental images (e.g., coal with mining, nuclear power with Chernobyl and cooling towers, natural gas with fires and pipes, wind with wind mills/turbines). Consistent patterns of relationships emerged among image evaluations, emotions, and beliefs about each of the energy sources such that coal and nuclear energy were viewed most negatively, natural gas was viewed more positively than coal and nuclear energy but less positively than wind power, with wind power considered the most favorable energy source. Affective image evaluations, emotions, and cognitive beliefs each explained levels of support for the energy sources.

Truelove’s (2012) study is an important pioneering piece of work in the study of affective images concerning energy transition. It is restricted in that it considered only four types of energy sources, and as a result, the complex multifaceted nature of energy transition seems somewhat underrepresented. As the study measured a variety of concepts in addition to affective images, such as emotions, cognitive beliefs, and policy support, it included a broad range of psychological variables for few transition components. Our approach is complementary in the sense that we aim to explore laypeople’s mental representation of the broad landscape of potential energy transition pathways. Specifically, we will include a broad range of energy transition pathway components but only one psychological measure of the mental representation, namely, affective images.

Affective images stem from personal experiences on the one hand, and social discourses and media reporting on the other (Smith and Joffe, 2013; Leiserowitz and Smith, 2017). It is because of this that social representations are likely to be shaped to some extent by socio-cultural contexts. Affective images are a highly sensitive measure of the public discourse about a topic, which makes it well suited to identify interpretative communities that differ in their conceptualizations of an issue (Fischer et al., 2012; Leiserowitz and Smith, 2017). This is exemplified by research on naysayers versus alarmists, two communities that interpret climate change through different lenses (Leiserowitz and Smith, 2017). Such communities are prone to engage in motivated reasoning, for example by seeking out information that confirms their views, and reinforcing each other in their interpretations; a process which may be amplified by social media (Williams et al., 2015).

## Research Aims

Our approach in studying public perceptions of energy transition extends prior research in several respects. First, in accordance with Böhm et al. (in press), this study conceptualizes energy transition as a multifaceted construct that comprises steps at different levels ranging from individual actions to policies, infrastructure, technologies, and international agreements. Rather than presenting respondents with a relatively small selection of energy transition pathway components, such as different energy sources, a comprehensive range of potential components is considered. As there is still little research on laypeople’s perceptions of energy transition in this broad sense, an exploratory approach is employed to tap into the mental representation of these components in the form of affective images (elicited through free associations and affective judgments) rather than studying specific beliefs or judgments (e.g., ascriptions of causes and consequences, attitudinal judgments).

Second, this study extends the existing literature by providing a comparison between two different cultural contexts, Norway and Germany, which differ in interesting ways with respect to their socio-political contexts and histories concerning energy systems (Arnold et al., 2016). For example, oil and hydroelectric power play important roles in Norway, the former as a source of employment and economic development and the latter as an energy source. The economic importance of fossil fuels in Norway blends with national identity and is in conflict with a general pro-environmental and climate-friendly national self-image. Germany is politically committed to the transition toward renewable energy (“Energiewende”), but has a long tradition of using coal as an energy source. Coal extraction is not only an economically important factor but also forms regional identities. Public engagement with energy in Germany is strongly shaped by opposition toward nuclear energy and high levels of environmental awareness. One further aim of our study is to map which content people mentally associate with various energy transition pathways in these two cultural contexts, and which affective evaluation they attach to these associations. Even though affective images can provide a rich source of tapping into and comparing meanings across individuals, groups, and cultures, studies addressing cultural context in affective imaging remain scarce (but see Lorenzoni et al., 2006).

## Methods

We collected data in a Norwegian and a German sample. We employed affective image analysis (see below) in both samples, a structured method to explore connotative and affective meanings. Affective image analysis uses free associations and evaluations of these associations. We will compare these two elements of affective image analysis across the two samples.

### Participants

Norwegian participants (*n* = 106; 81 women, 25 men; *M*_age_ = 23.7, *SD*_age_ = 3.67, age range: 19–39) were students at the University of Bergen in Norway. They were recruited via student Facebook groups associated with the Faculty of Psychology. Most participants (*n* = 102) were enrolled in a psychology program (predominantly clinical psychology or work and organizational psychology); the remaining four participants came from biology, law, social economics, and theater science, respectively. A majority (*n* = 63) indicated that they had heard the term energy transition before participating in the study, while the remaining 43 participants responded that this was not the case.

German participants (*n* = 125; 88 women, 24 men, 1 recorded non-binary, 12 did not respond to the gender question; *M*_age_ = 22.0, *SD*_age_ = 3.32, age range: 18–35, 15 did not report their age) were students at the Leuphana University of Lüneburg in Germany. They were recruited in psychology lectures. Most (*n* = 72) studied psychology, 35 marketing and management, 2 a combination of environmental sciences and psychology, and 1 other (individual studies); 15 did not indicate their study program. All indicated that they had heard the term energy transition before participating in the study.

Participants from both countries were informed about the topic and aims of the study, the anonymity of their answers, and the right to withdraw at any time from their participation. Consent of the participants was obtained by virtue of survey completion.

### Materials

#### Energy Transition Pathway Components

The main stimulus material consisted of 25 terms that describe actions that can be taken as part of a strategy toward sustainable ways of producing and using energy. We aimed to select a set of components that would cover a broad range of possible actions and include those that are relevant in the public’s mind as well as from a scientific and political perspective. We based the selection on four sources: (a) general desk research on the issue of energy transition, (b) desk research of the psychological and social science literature to identify environmental behaviors and policy options used in previous studies, (c) pilot interviews with students from the same target population as the participants in the current study, and (d) interviews with experts from the climate and political sciences. The components correspond in part to those used by Böhm et al. (in press). They comprise actions on three broad levels: individual actions (e.g., using public transportation), political actions (e.g., international agreements), and technologies (e.g., carbon capture and storage). They also included two types of action that are distinguished in the environmental psychology literature (e.g., Gardner and Stern, 2008), namely curtailment (e.g., energy saving) and efficiency (e.g., energy efficient household articles). A complete list of the 25 components is given in **Table 1**.

**Table 1:** The 25 energy transition pathway components used in this study.

Label	Energy transition pathway component
appliances	Energy efficient home appliances (e.g., light bulbs)
ccs	Carbon capture and storage
compensate	Climate compensation (e.g., when buying flights)
e.cars	Electric cars
educ	Environmental education (e.g., in school, at work)
engage	Political engagement
flights	Avoid long flights
houses	Energy efficient houses (e.g., geothermal heating)
hydro	Hydropower
int.agree	International agreements (e.g., on carbon emissions)
int.trade	International trade with carbon offsets
it	Information technologies (e.g., monitor home energy use)
nuclear	Nuclear power
pub.trans	Public transportation
regulate	Regulations (e.g., laws to reduce sales of fossil fuel cars)
saving	Energy saving (e.g., turn down heating)
science	Science
sharing	Sharing economy (e.g., carpooling)
solar	Solar panels
subsidy	Subsidies (e.g., for renewable energy)
tax	Taxes (e.g., on carbon intensive goods and services)
urban	Urban planning (e.g., car free zones)
vegetar	Vegetarian food
walking	Walking and cycling
wind	Wind farms

#### Measures

We followed a method described by Leiserowitz and colleagues (Leiserowitz, 2006; Lorenzoni et al., 2006; Leiserowitz and Smith, 2017) as affective image analysis (see also Pfister et al., 2000, for an application to the public perception of genetic engineering). The basic idea is to tap into people’s mental images and affective connotations concerning some issue by having them first generate one or more free associations to the issue in question and then asking them to evaluate the affective valence of their own free associations. The content of the free association gives an indication of the mental images that people associate with the issue; the evaluation of the free associations reflects their affective connotations on a dimension of positive to negative valence. Mental images and their affective evaluation constitute the two elements of affective images (Leiserowitz and Smith, 2017).

##### Free associations

For each energy transition pathway component, participants were asked to briefly describe the first thought that came to their minds with respect to this component. They were instructed to answer spontaneously and swiftly but without rushing. They gave their responses in writing in a free text field in the questionnaire.

##### Evaluation of free associations

After having completed the free association task, participants were requested to go through their free associations again and to indicate for each whether they considered it something positive, or negative, or neutral (neither positive nor negative). Responses were coded as +1, -1, or 0, respectively.

##### Background variables

At the end of the questionnaire, participants were asked whether they had heard the term energy transition before participating in the study (yes/no). They were also asked to indicate their age, gender, and study program.

#### Coding of Free Associations

The free associations were content analyzed (Bos and Tarnai, 1999) in order to capture the content of the mental images. We developed a coding scheme in a bottom-up manner in the following steps: first, four individuals (two of the authors, plus two research assistants from the same target population as the respondents, i.e., university students) looked at the responses independently and came up with a proposal for categories. These four proposals were then merged by discussion and developed into a common coherent category system. We described this category system in writing, giving for each category a definition and examples. The resulting coding scheme consists of five superordinate categories, each being divided in subcategories; the complete list of categories is given in **Table 2**. The complete coding scheme including coding instructions and examples of responses for each category is provided in the **Supplementary Material**. The five superordinate categories are: (a) requirements (i.e., the response indicates that the component will not work in isolation but requires some additional action; subcategories refer to requirements at the level of international politics, national politics, or individual life styles), (b) consequences (i.e., the response refers to potential positive or negative consequences of the component; subcategories refer to consequences for individuals, society, or the environment), (c) evaluation (i.e., the respondent expresses an evaluation of the component, for example concerning its feasibility or importance), (d) prevalence (i.e., the response refers to the prevalence of the component, indicating how widespread, or rare, it is; for example indicating that the component applies only to certain people), and (e) remnant categories (e.g., mere descriptions of the component, when the response stated some descriptive aspect of the component such as that electric cars are electric).

**Table 2:** Distribution of the free associations across the categories of the coding scheme, aggregated across all energy transition pathway components, for Norwegian and German sample (percent).

	Codes			Category	Percentages Norway			Percentages Germany		
Label	Level 1	Level 2	Level 3		Level 1	Level 2	Level 3	Level 1	Level 2	Level 3
**R1**	**1**			**Requirements**	**15.66**			**15.50**		
R1.0		10		*No specification*		0			0.91	
R1.1		11		Requirement on international level		1.32			1.30	
R1.1.0			110	*No specification*			0.11			0.25
R1.1.1			111	Need for international agreements			0.72			0.53
R1.1.2			112	Need for monitoring targets			0.49			0.53
R1.2		12		Requirement on the level of national policies		9.51			9.77	
R1.2.0			120	*No specification*			0.75			1.86
R1.2.1			121	Regulation via incentives			2.19			2.57
R1.2.2			122	Regulation via punishments			0.79			0.88
R1.2.3			123	Need for facilitation (available infrastructure)			3.66			2.64
R1.2.4			124	Need to increase knowledge (fund research)			2.11			1.83
R1.3		13		Requirement on the level of the citizens within a society		4.83			3.51	
R1.3.0			130	*No specification*			0.04			0.07
R1.3.1			131	Need to change behavior/lifestyles			1.28			1.41
R1.3.2			132	Need to change attitudes/values			0.26			0.18
R1.3.3			133	Need for collective action			2.00			0.84
R1.3.4			134	Need to increase awareness			1.25			1.02
**R2**	**2**			**Consequences**	**14.26**			**13.00**		
R2.0		20		*No specification*		0			1.09	
R2.1		21		Personal consequences		6.45			6.11	
R2.1.0			210	*No specification*			0.11			0.70
R2.1.1			211	Personal time resources			0.30			0.18
R2.1.2			212	Personal financial resources			2.49			2.35
R2.1.3			213	Personal comfort			1.47			1.37
R2.1.4			214	Personal social interactions			0.11			0.14
R2.1.5			215	Personal health effects			1.43			1.16
R2.1.6			216	Personal freedom			0.53			0.21
R2.2		22		Societal consequences		1.02			0.81	
R2.2.0			220	*No specification*			0.04			0.21
R2.2.1			221	Social risks			0.08			0.07
R2.2.2			222	Social justice			0.91			0.53
R2.3		23		Environmental consequences		6.79			4.99	
R2.3.0			230	*No specification*			3.55			0.25
R2.3.1			231	Environmental pollution			0.83			1.51
R2.3.2			232	Environmental preservation			1.32			2.85
R2.3.3			233	Environmental aesthetics			1.09			0.39
**R3**	**3**			**Evaluation**	**48.98**			**49.23**		
R3.0		30		*No specification*		0.60			1.62	
R3.1		31		Evaluation concerning feasibility		3.36			4.85	
R3.2		32		Evaluation concerning effectiveness		5.36			2.11	
R3.3		33		Evaluation concerning importance		10.83			13.63	
R3.3.0			330	*No specification*			8.72			11.52
R3.3.1			331	Importance for the present			0.30			0.25
R3.3.2			332	Importance for the future			1.81			1.86
R3.4		34		Expression of skepticism		4.00			7.77	
R3.4.0			340	*No specification*			1.92			5.87
R3.4.1			341	Skepticism toward underlying intentions			1.55			1.83
R3.4.2			342	Skepticism toward the scientific bases			0.53			0.07
R3.5		35		Expression of affective valence		16.98			9.28	
R3.5.0			350	*No specification*			0.19			
R3.5.1			351	Positive affect			14.08			6.99
R3.5.2			352	Negative affect			2.72			2.28
R3.6		36		Expression of conflicting aspects		7.85			9.98	
R3.6.0			360	*No specification*			0.15			1.16
R3.6.1			361	Conflict between different impacts			7.66			8.75
R3.6.2			362	Conflict between different generations			0.04			0.07
**R4**	**4**			**Prevalence**	**2.38**			**8.57**		
R4.0		40		*No specification*		0.60			4.32	
R4.1		41		Prevalence with respect to personal actions		1.17			3.65	
R4.1.0			410	*No specification*			0.19			0.46
R4.1.1			411	Respondent is already doing it			0.75			2.39
R4.1.2			412	Respondent lacks motivation			0.23			0.81
R4.2		42		Prevalence among certain social groups		0.60			0.60	
R4.2.0			420	*No specification*			0.19			0.25
R4.2.1			421	Prevalence among certain subcultures			0.19			0.28
R4.2.2			422	Prevalence among demographic groups			0.23			0.07
**R5.0**				**Remnant Categories**	**18.72**			**13.70**		
R5.1		51		Mere description		11.02			10.01	
R5.2		52		Non-codeable response		2.68			2.28	
R5.3		53		Don’t know response		5.02			1.41	
				**Sum**	**100**	**100**	**71.36**	**100**	**100**	**71.40**

*Percentages are based on n = 2650 responses in Norway and n = 2846 responses in Germany. Level 1 categories are in bold face. Subcategories add up to the next higher superordinate category*.

The responses differ quite strongly in specificity. For example, some people said something like “that’s important” or “that’s good”; or, with respect to responses falling in the category of requirements, some participants said something like “won’t work on its own” very generally; while others were more specific and said something like “important to have binding agreements that include sanctions if they are broken.” In order to capture such differences in specificity, the coding scheme contains categories at three levels of specificity (see **Table 2**). Responses were coded at the most specific category possible. Superordinate categories were used when the response did not give more specific information to assign it to a subcategory (in **Table 2** listed as codes labeled as *no specification*) or if something specific was said that did not match any of the available subcategories.

In both samples, two university students coded the free associations. These were Norwegian native speakers for the Norwegian data and German native speakers for the German data. First, the two coders coded the responses independently. They were then asked to go through the responses on which they had disagreed and discuss whether they could solve the disagreement.

The Norwegian sample generated 2650 free associations. In their independent coding, coders agreed in 69.3% of these responses, Cohen’s Kappa = 0.674, *p* < 0.001, considering all three levels of the coding scheme. At Level 1, the two coders agreed in 80.3% of the responses, Kappa = 0.712, *p* < 0.001. After having discussed their disagreements, coders assigned a mutual code to all of the 2650 responses.

The German sample generated 2946 free associations. Intercoder-agreement was lower in the German than in the Norwegian sample. In their initial independent coding, the two coders agreed in 47.0% of the responses, Cohen’s Kappa = 0.446, *p* < 0.001, considering all three levels of the coding scheme. At Level 1, agreement was 71.4%, Kappa = 0.61, *p* < 0.001. After discussing disagreements, the coders assigned a mutual code to 2846 of the responses.

### Procedure

In Norway, data collection was done in a computer lab. Each participant was seated at an individual computer that was shielded by partitioning walls at the sides and at the front. Computer lab sessions were run in groups of 16 to 29 participants. All materials were presented and data collected via a computer-based survey (programmed in an online tool called Explorable^1^). The data reported in this paper were collected at the beginning of a larger survey; the entire lab sessions lasted on average 45 min. For the entire survey, participants received a gift voucher worth NOK 200.00 (ca. EUR 21.00) as an incentive for participating.

In Germany, data were collected by means of a paper-and-pencil questionnaire that was distributed at the end of lectures. The questionnaire consisted only of the free association and evaluation tasks (plus the background variables). Participants needed on average 20 min to fill in the questionnaire; they received a chocolate bar and a ballpoint pen as an incentive for their participation.

Participants were informed that the study dealt with the question of how people think and feel about various steps that can be taken as part of energy transition, which was defined as long-term changes in energy systems that aim at fostering a more sustainable society. The energy transition pathway components were then presented, each followed by an open text field on the computer screen (Norway) or a blank space on the paper questionnaire (Germany) for participants to fill in their free associations. Each participant received the components in one of two random orders. After participants had filled in their free associations, they were asked to evaluate them. In the computer-based procedure in Norway, participants were presented with their own free associations that they had entered before. In the paper-and-pencil based procedure in Germany, participants were asked to turn back in their questionnaire to evaluate their free associations. At the end of the questionnaire, participants had the opportunity to leave comments. Upon having completed the questionnaire, participants were thanked and received their incentive.

## Results

We will first focus on the content of the free associations and report the distributions of the free associations across the categories of the coding scheme. We then report the results concerning participants’ evaluations of their free associations. All statistical analyses were done using the R statistical environment (R Core Team, 2018).

### Free Associations

#### Aggregated Distribution of Free Associations

**Table 2** lists for each category of the coding scheme what percentage of the free associations falls into that category, aggregated across all energy transition pathway components. The two distributions for the Norwegian and German data are remarkably similar. By far the most frequent type of free association, accounting for slightly less than 50% in both samples (48.98% in Norway and 49.23% in Germany), is a general evaluation of the energy transition pathway component in response to which the free association was generated. The most frequent subcategories are an evaluation of the component’s importance (e.g., “that would matter a lot”; 10.83 and 13.63% for Norway and Germany, respectively) and an expression of affective valence (e.g., “that’s a good thing”; 16.98%, 9.28%), followed by an expression of conflicting aspects, especially conflicting impacts (e.g., “good for the environment, but expensive”; 7.66%, 8.75%).

The second most frequent categories, by a notable margin, are requirements and consequences, accounting for 13.00–15.66% of the free associations. Requirement means that the participant expressed some requirement needed to make a component work. These requirements often referred to national policies (e.g., necessary regulation or infrastructure; 9.51%, 9.77%) or, less often, to something required of the citizens in a society (e.g., lifestyle changes; 4.83%, 3.51%). Consequences means that the free association referred to a consequence of the energy transition pathway component, most often to personal consequences such as financial costs (6.45%, 6.11%) or to environmental consequences (6.79%, 4.99%).

The least frequent type of association referred to the prevalence of a component (2.38%, 8.57%). The remnant category comprises associations where the respondent either merely rephrased the component (the most frequent remnant category; 11.02%, 10.01), or responses that fit none of the categories (2.68%, 2.28%) or don’t know responses (5.02%, 1.41%).

#### Free Associations to Individual Energy Transition Pathway Components

In order to explore which types of free associations were generated with respect to which energy transition pathway component, we will consider only Level 1 codes from the coding scheme; the frequencies of the subcategories get too low when broken down across individual components. **Tables 3, 4** show the percentage distributions of Level 1 code categories across components for the Norwegian and the German sample, respectively.

**Table 3:** Distribution of Level 1 codes for all energy transition pathway components, Norwegian data (percent).

Energy transition pathway component	Level 1 codes				
	Requirements	Consequences	Evaluation	Prevalence	Remnant
appliances	0.72	0.72	2.26	0.04	0.26
ccs	0.15	0.11	1.32	0.00	2.42
compensate	0.19	0.45	1.92	0.00	1.43
e.cars	0.34	1.09	1.55	0.15	0.87
educ	1.36	0.04	2.15	0.04	0.42
engage	0.60	0.04	2.57	0.26	0.53
flights	0.68	0.60	2.42	0.11	0.19
houses	0.60	0.38	1.89	0.23	0.91
hydro	0.45	0.75	1.25	0.11	1.43
int.agree	1.09	0.15	2.23	0.00	0.53
int.trade	0.34	0.60	2.19	0.00	0.87
it	1.28	0.19	1.70	0.00	0.83
nuclear	0.11	0.53	2.45	0.00	0.91
pub.trans	1.66	0.79	1.17	0.19	0.19
regulate	0.49	0.38	2.26	0.04	0.83
saving	0.91	1.02	1.28	0.26	0.53
science	0.38	0.11	2.57	0.00	0.94
sharing	0.60	0.60	2.19	0.08	0.53
solar	0.45	0.42	1.96	0.15	1.02
subsidy	0.42	0.15	2.49	0.00	0.94
tax	0.68	0.45	2.34	0.08	0.45
urban	0.60	0.72	2.23	0.08	0.38
vegetar	0.75	1.06	1.66	0.23	0.30
walking	0.60	1.96	1.02	0.23	0.19
wind	0.19	0.94	1.92	0.11	0.83

**Sum**	**15.66**	**14.26**	**48.98**	**2.38**	**18.72**

*Percentages are based on n = 2650 responses. Abbreviations of energy transition pathway components are explained in **Table 1**.*

**Table 4:** Distribution of Level 1 codes for all energy transition pathway components, German data (percent).

Energy transition pathway component	Level 1 codes				
	Requirements	Consequences	Evaluation	Prevalence	Remnant
appliances	0.98	0.60	1.76	0.53	0.35
ccs	0.25	0.25	0.49	0.04	0.70
compensate	0.53	0.49	1.51	0.25	0.88
e.cars	0.42	0.60	2.35	0.35	0.67
educ	0.63	0.07	2.28	0.88	0.49
engage	0.63	0.39	1.83	0.88	0.32
flights	0.14	0.70	2.71	0.49	0.28
houses	0.56	0.74	2.04	0.35	0.46
hydro	0.35	0.53	2.00	0.25	0.74
int.agree	0.91	0.04	2.28	0.04	0.60
int.trade	0.35	0.25	1.86	0.00	0.49
it	0.35	0.56	2.35	0.18	0.63
nuclear	1.23	0.28	2.18	0.00	0.67
pub.trans	1.58	0.77	1.19	0.28	0.46
regulate	0.70	0.32	2.32	0.07	0.74
saving	1.02	0.46	1.37	0.95	0.56
science	0.70	0.25	1.79	0.11	1.23
sharing	0.39	0.91	2.11	0.67	0.32
solar	0.63	0.42	1.62	0.56	0.95
subsidy	0.63	0.25	2.35	0.14	0.42
tax	0.53	0.49	2.46	0.00	0.35
urban	0.49	0.49	2.57	0.11	0.46
vegetar	0.42	0.98	2.14	0.60	0.18
walking	0.60	1.44	1.41	0.77	0.18
wind	0.46	0.74	2.25	0.11	0.60

**Sum**	**15.50**	**13.00**	**49.23**	**8.57**	**13.70**

*Percentages are based on n = 2846 responses. Abbreviations of energy transition pathway components are explained in **Table 1**.*

Again, the two samples show a very similar pattern; this is indicated numerically by a high correlation between the Norwegian and the German frequencies across the cells of the cross-tabulation of Level 1 codes with energy transition pathway components (i.e., across the cells of **Tables 3, 4**), *r* = 0.86, *p* < 0.001.

**FIGURE 1:**
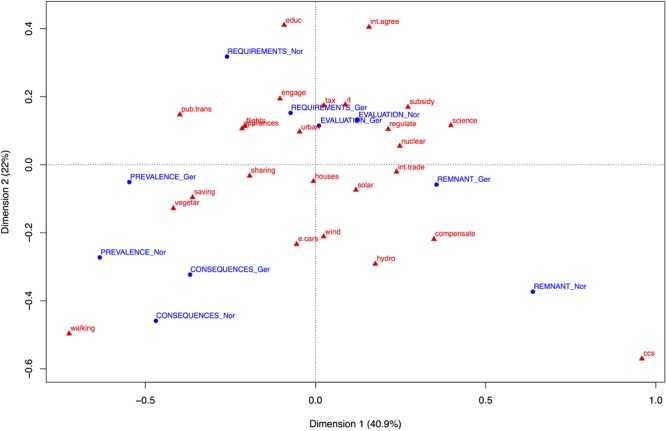
Correspondence analysis plot of Level 1 code categories cross-tabulated with energy transition pathway components; common analysis of Norwegian and German data. Energy transition pathway components are labeled in lower-case letters and red font. Level 1 codes are labeled in blue font and upper-case letters; labels of Level 1 codes for the Norwegian sample end on “_Nor”, those for the German sample on “_Ger”. See **Table 1** for labels of the energy transition pathway components.

The relationship between type of free association, as captured by Level 1 codes, and energy transition pathway components was explored by means of a correspondence analysis (Greenacre, 1984, 1993), which is depicted in **Figure 1**. Norwegian and German data were analyzed in a common analysis. The correspondence analysis provides a graphical representation of the association between Level 1 codes and components. We selected the two-dimensional configuration for interpretation, yielding a cumulative principal inertia = 62.95% (see **Figure 1**). The distances among the components in this plot reflect how similar their distributions are across the Level 1 codes; components that are located close to each other have elicited a similar pattern of free associations. Likewise, the distances among Level 1 codes reflect resemblance of their distributions across components; Level 1 codes that are located close to each other have been generated in similar patterns in response to the energy transition pathway components.

The corresponding Level 1 codes for the Norwegian and the German sample are located in close proximity to each other. Thus, the energy transition pathway components generated similar patterns of free associations in the two samples; as was already indicated by the high correlation between the two samples concerning Level 1 code frequencies across components. In order to interpret which types of free associations were generated for which energy transition pathway components, imagine for each component a line that connects the component with the origin of the coordinate system. The projection of a Level 1 code onto this imagined line indicates how closely this type of free association relates to the component.

Evaluation is located close to the origin, which indicates that evaluations are not specific for any particular component; they are generated frequently across all components. Evaluations are the most typical free association overall. Components in the lower left quadrant, especially walking, generated associations concerning consequences and prevalence. The remnant category is most closely associated with carbon capture and storage and also with carbon compensation; especially in Norway also with hydropower. Public transportation, environmental education, and international agreements elicit associations that reflect that people see them as needing further requirements, particularly in the Norwegian sample. Energy efficient houses is the energy transition pathway component that is most closely at the origin of the configuration, which indicates that it is the component whose pattern of free associations is most similar to the average pattern across all components. This could imply that energy efficient houses are the most prototypical energy transition pathway component in laypeople’s minds.

The horizontal dimension as a whole separates individual action on the left side (e.g., walking, vegetarian food, energy saving, public transport) from political-societal actions (in the upper half; e.g., science, subsidies, regulation) and technologies (in the lower half; e.g., CCS, nuclear-, solar-, hydropower) on the right. Individual actions are associated with prevalence, consequences, and requirements (especially Norway); political-societal actions and technologies elicited primarily descriptive associations (the predominant remnant category) and also more evaluations than average (in Norway).

### Evaluation of Free Associations

Participants’ evaluations of their own free associations as either positive, neutral, or negative are summarized in **Table 5**. Concerning the sample sizes, note that the Norwegian sample has no missing values; all participants provided a free association to each and every energy transition pathway component, and also evaluated each and every of their free associations. In the German sample, in contrast, there are missing values in both the free associations and their evaluations. We assume that this difference was brought about by the different data collection methodologies, computer-based versus paper-and-pencil questionnaire, that were applied in the two samples.

**Table 5:** Average evaluation of free associations per energy transition pathway component.

	Norwegian sample					German sample				
Energy transition pathway component	*n*	*M*	*SD*	95%CI lower limit	95%CI upper limit	*n*	*M*	*SD*	95%CI lower limit	95%CI upper limit
appliances	106	0.75	0.49	0.66	0.85	110	0.75	0.53	0.66	0.85
ccs	106	–0.04	0.60	–0.15	0.08	54	–0.67	0.48	–0.79	–0.54
compensate	106	0.01	0.79	–0.14	0.16	102	0.15	0.84	–0.02	0.31
e.cars	106	0.70	0.59	0.59	0.81	114	0.35	0.81	0.20	0.50
educ	106	0.84	0.46	0.75	0.93	114	0.59	0.76	0.45	0.73
engage	106	0.55	0.66	0.42	0.67	62	0.29	0.88	0.07	0.51
flights	106	–0.23	0.78	–0.38	–0.08	78	–0.35	0.83	–0.53	–0.16
houses	106	0.66	0.57	0.55	0.77	112	0.64	0.67	0.52	0.77
hydro	106	0.82	0.43	0.74	0.90	57	0.30	0.78	0.10	0.50
int.agree	106	0.35	0.77	0.20	0.50	69	–0.16	0.87	–0.36	0.05
int.trade	106	0.02	0.85	–0.14	0.18	94	–0.56	0.60	–0.68	–0.44
it	106	0.59	0.57	0.49	0.70	108	0.40	0.77	0.25	0.54
nuclear	106	–0.54	0.71	–0.67	–0.40	113	–0.58	0.75	–0.71	–0.44
pub.trans	106	0.54	0.76	0.39	0.68	114	0.54	0.73	0.41	0.68
regulate	106	0.54	0.72	0.40	0.67	109	0.24	0.87	0.08	0.40
saving	106	0.52	0.75	0.38	0.66	114	0.77	0.53	0.67	0.87
science	106	0.83	0.42	0.75	0.91	109	0.72	0.49	0.62	0.81
sharing	106	0.56	0.60	0.44	0.67	114	0.82	0.50	0.73	0.92
solar	106	0.70	0.57	0.59	0.81	59	0.56	0.73	0.37	0.74
subsidy	106	0.75	0.47	0.66	0.85	103	0.60	0.72	0.46	0.74
tax	106	0.27	0.75	0.13	0.42	66	0.05	0.85	–0.16	0.25
urban	106	0.62	0.62	0.50	0.74	63	0.29	0.81	0.09	0.49
vegetar	106	0.54	0.71	0.40	0.67	114	0.55	0.67	0.43	0.67
walking	106	0.83	0.49	0.74	0.92	114	0.83	0.48	0.75	0.92
wind	106	0.70	0.57	0.59	0.81	64	0.45	0.85	0.24	0.66

*Abbreviations of energy transition pathway components are explained in **Table 1**.*

**FIGURE 2:**
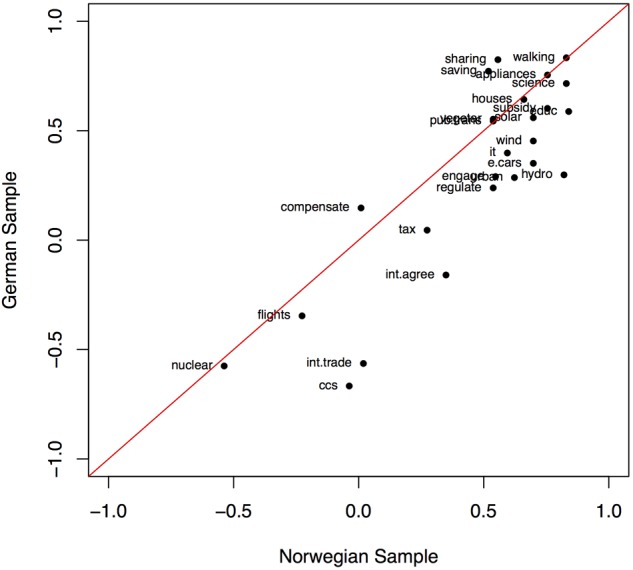
Scatterplot of participants’ evaluations of their own free associations, averaged across participants for each energy transition pathway component. Means for the Norwegian sample are plotted along the horizontal axis, those of the German sample along the vertical axis. See **Table 1** for labels of the energy transition pathway components.

A graphical depiction of the average evaluations of the free associations for each energy transition pathway component is shown in **Figure 2**. The plot illustrates that the Norwegian and German mean evaluations across the components are highly correlated, *r* = 0.85, *p* < 0.001. Nuclear power, carbon capture and storage, avoid long flights, and international trade with carbon offsets are the components that elicited the most negatively evaluated free associations in both samples. Free associations to climate compensation such as buying carbon offsets for flight tickets, to international agreements, and to taxes generated slightly positively evaluated free associations in both countries. Looking at the top four components with the most positively evaluated free associations in each sample, we find that *educ, walking, science*, and *hydro* are the top components for the Norwegian sample, and that *walking, sharing, saving*, and *appliances* are the top components for the German sample. Whereas Norwegians seem to have favorable associations toward general political strategies (*educ, science*) and, maybe not surprisingly given its prevalence in the country, the use of hydropower, Germans are more favorable toward individual activities such as participation in the sharing economy (e.g., car pooling) or saving energy (e.g., turning down the heating).

If the free associations for a component are evaluated equally in both samples, the component lies on the diagonal. This is the case only for a few of the components. There is a general tendency of Norwegian participants to evaluate their free associations to the components more positively than German participants do. Exceptions are climate compensations, sharing economy, and energy saving, whose free associations are more positively evaluated in the German than in the Norwegian sample.

## Discussion

This study explored which mental images and affective evaluations laypeople associate with various energy transition pathway components, as have been described in the beginning of this paper. A remarkable result is the similarity between Norwegian and German participants despite differences in the socio-political contexts and traditions concerning energy. Considering studies showing that social representations are at least partly shaped by the socio-cultural context (Smith and Joffe, 2013) and that affective images are learned from experience (Leiserowitz, 2006; Lorenzoni et al., 2006), we expected that the energy transition pathway components presented would be associated with divergent affective images in the two study samples. This turned out to not be the case; the overall distributions of the free associations across the categories of our coding scheme were virtually identical. Both samples also associated very similar patterns of mental images with the different energy transition pathway components, as illustrated in the correspondence analysis. The fact that average evaluations of the free associations for the individual components were highly correlated provides further grounds to establish that the mental representation of the energy transition pathway components were very similar among Norwegian and German participants.

We drew quite homogeneous samples; namely, university students at both locations. While this homogeneity facilitates comparisons, it may have minimized variation in social and educational backgrounds. Some of the differences that exist between Norway and Germany concerning these countries’ socio-political energy contexts are presumably experienced more intensely in other socio-economic and professional groups than university students. A recent study showed, for instance, that employees in the Norwegian oil and gas industry tend to show less support for policies that restrict the production of fossil fuels than the larger population (Tvinnereim and Ivarsflaten, 2016). It cannot be ruled out that the choice of samples may have precluded larger variations in affective images to show up; still, the consistency of the results across samples is suggestive and raises confidence that the results are not merely random but may be descriptive of the underlying, albeit homogeneous, population of university students.

The most frequent type of free association was a general evaluation of the energy transition pathway component in response to which the association was generated. This most often referred to the level of importance assigned to each component, or to an affective evaluation of the component as something good or bad. Less often did the participants express that the component entails conflicting aspects, positive and negative, mostly referring to conflicting good and bad impacts. Other (less frequent) evaluations concerned the feasibility or effectiveness of the component or expressed some skepticism, for example concerning the trustworthiness of involved actors as well as their intentions. At a large interval from these evaluations, the second most frequent types of association are requirements needed to make a component work (usually some requirement at the level of national policies or individual actions), and consequences, typically personal consequences affecting finances or comfort or environmental consequences. About equally frequent as requirements and consequences were mere descriptions of the component or some aspect of it.

Rather than mentioning any detail that would hint at an elaborate mental representation of the components, the free associations generated by the participants suggest that knowledge about the presented pathway components is rather vague and unspecific. This matches prior studies indicating that people often hold a general pollution model according to which anything that pollutes the environment is also bad for the climate (Bord et al., 1998). People then tend to apply a corresponding good environmental practice heuristic assuming that all actions that are good for the environment will also help mitigate climate change (Read et al., 1994). Our results suggest that energy transition is processed on a similarly general and unspecific level.

Nevertheless, people express clear evaluations of the components as good or bad, important or unimportant, effective or ineffective. One might assume that strong evaluations are based on knowledge; that people become more opposed or supportive of an issue, the more they know about it. The positive relationship between knowledge and polarization that has been found in the climate change literature supports this assumption (Kahan et al., 2012; Guber, 2013), though it has been argued that communicating scientific facts can neutralize polarization (van der Linden et al., 2017). What our results seem to indicate, however, is that relatively strong evaluations are triggered in the absence of a correspondingly strong knowledge base. A similar disconnect between knowledge and evaluation has been documented for the public perception of genetic engineering (Pfister et al., 2000). In terms of dual systems theories (e.g., Kahneman, 2011), energy transition pathways seem to be processed in an automatic and intuitive rather than a deliberative manner. That requirements are among the most frequent associations suggests that people hold a systemic view of energy transition, where one action is not sufficient, but the conjunction of many elements is required to bring about positive effects. If attempting to characterize the overall mental model of energy transition that emerges from our data, there appears to be a vague and intuitive understanding of a systemic interaction of many components, with the most relevant consequences being those for individual citizens and the environment. Similar results were found in an international qualitative study on social representations of climate and energy (Fischer et al., 2012), which found across five European countries the people see such issues not as isolated phenomena but contextualize them in a broader general framework of energy-related issues.

When considering which types of association are generated in response to which energy transition pathway components, there seems to be a divide between individual actions, on one side, and socio-political actions and technologies, on the other side. This may indicate that people recognize the collective nature of energy transition, in addition to seeing individual behaviors as embedded in the societal context. It seems that with respect to individual actions people are most preoccupied with whether or not other people will join in and adopt the behavior (prevalence), what the personal consequences of the behavior are, whether the behavior is effective (environmental consequences), and that individual behavior depends on contextual conditions (requirements), such as the availability of public transport or other infrastructures. These three types of action seem to reflect a fundamental distinction in laypeople’s thinking about energy transition (for similar findings, see Böhm et al., in press).

By far the most negatively evaluated energy transition pathway component was nuclear power, whereas renewables such as solar-, wind-, and hydropower were located at the positive pole of evaluation. This resembles the pattern reported in another study that employed an affective image analysis with an explicit focus on energy sources (Truelove, 2012). Other large-scale survey research has also found that nuclear power tends to be evaluated more negatively than renewable energy sources (Steentjes et al., 2017), even though support for nuclear power as a climate mitigation strategy can show large variation across countries (Doran et al., 2018). One reason for why renewables are evaluated rather positively could be that people associate these energy sources with the future (Fischer et al., 2012). Also very positively evaluated were associations to individual actions such as walking and cycling, policies such as subsidies and regulation, and science. These are again options that have been found to be positively regarded by the public in more comprehensive survey research (e.g., Bostrom et al., 2012). While we did not measure support for these components, other studies found a positive relationship between affective images and behavioral measures such as policy support (Leiserowitz, 2006; Smith and Leiserowitz, 2012; Truelove, 2012) and thus suggest that the positively evaluated components would also be likely to be supported.

An obvious limitation of this study concerns the small samples. Both samples were convenience samples, drawn from accessible pools of university students that cannot serve for drawing inferences regarding the wider public in each of the two countries. Although we do not claim to provide an international comparison, the results are very similar across the two countries, which suggests some stability. We therefore hope that our results have heuristic value and can guide future research in the study of the mental representation of energy transition pathways. We believe that the contents of the free associations as identified in our coding scheme give a good reflection of people’s concerns with respect to different energy transition pathways. We also believe that the cognitive structure of the components that emerged from the patterns of free associations connected to them and from the affective evaluation of these associations are worthy of further exploration in systematic survey and experimental research. The labor intense coding of the free associations precluded the use of larger samples in the present study. However, the emergence of new computer-based automated linguistic analysis techniques, such as structural topic models, may open up new avenues for collecting and analyzing free responses in large-scale surveys (see e.g., Tvinnereim and Fløttum, 2015).

## Ethics Statement

This empirical study complied with the Norwegian Social Science Data Services (NSD) privacy regulations and the ethical principles of research by the National Committee for Research Ethics in the Social Sciences and the Humanities (NESH). Formal approval from NSD was not sought, as the collected data material was anonymous, see www.nsd.uib.no/ personvernombud/en/notify/index.html.

## Data Availability

The raw data supporting the conclusions of this manuscript will be made available by the authors, without undue reservation, to any qualified researcher.

## Author Contributions

GB and RD contributed conception and design of the study. GB and H-RP performed the statistical analyses. GB wrote the first draft of the manuscript. GB, RD, and H-RP wrote sections of the manuscript. All authors contributed to manuscript revision, read and approved the submitted version.

## Conflict of Interest Statement

The authors declare that the research was conducted in the absence of any commercial or financial relationships that could be construed as a potential conflict of interest.

## Footnotes

1 https://explorable.com/
