# Supplementary material for: Laypeople’s Affective Images of Energy Transition Pathways
Source: Front Psychol. 2018 Oct 10;9:1904. doi: 10.3389/fpsyg.2018.01904 (PMC6191516; doi:10.3389/fpsyg.2018.01904)
Supplement: Supplementary file 1 [file Table_1.docx]

Supplementary Material

Lay people’s affective images of energy transition pathways

Gisela Böhm*, Rouven Doran, Hans-Rüdiger Pfister

*** Correspondence:** Gisela Böhm: [gisela.boehm@uib.no](mailto:gisela.boehm@uib.no)

# Coding Instructions for Free Associations

We ask for your help in categorizing responses that were given by participants in a study on energy and sustainability. The aim of the study is to find out what people think about energy transition (i.e., changes in the energy system that are taken in order to contribute to a more sustainable society). We presented a list of terms to our respondents, which describe steps that may be taken as part of energy transition. For each term, we asked respondents what the first thing was that came to their mind when hearing the term.

Here is the list of terms:

- Urban planning (e.g., car free zones)
- Regulations (e.g., laws to reduce sales of fossil fuel cars)
- Energy saving (e.g., turn down heating)
- Nuclear power
- Sharing economy (e.g., carpooling)
- Environmental education (e.g., in school, at work)
- Solar panels
- Hydropower
- Energy efficient houses (e.g., geothermal heating)
- Information technologies (e.g., monitor home energy use)
- Vegetarian food
- Wind farms
- Energy efficient home appliances (e.g., light bulbs)
- Subsidies (e.g., for renewable energy)
- Climate compensation (e.g., when buying flights)
- Carbon capture and storage
- Electric cars
- Science
- Taxes (e.g., on carbon intensive goods and services)
- International agreements (e.g., on carbon emissions)
- Avoid long flights
- International trade with carbon offsets
- Public transportation
- Political engagement
- Walking and cycling

The terms were presented one-by-one [Norway: on a computer screen / Germany: in a questionnaire]. Beneath each term was a text field. Respondents were asked:

**For each of these steps, please describe briefly the first thing that comes to your mind when you think of it.**

We want you to categorize the responses to this question according to a coding scheme, which you find below.

## Coding Scheme

We provide you with a detailed coding system, which we ask you to use in order to categorize a response. You will see that the responses differ a lot in specificity. For example, some people just say “Won’t work on its own” very generally; others are more specific and say something like “Important to have binding agreements that include sanctions if they are broken”. Accordingly, the categories in our coding system differ in their specificity in order to capture these differences. That is, the categories have subcategories.

In the table below you find for each category a description, 1-3 examples of responses that would fall into this category, and a code (a number).

Study this table carefully.

When coding the responses, use your best judgment as to what the respondent meant when giving the response. Assign each response to exactly one category – not more! If you think that a response contains more than one aspect and thus qualifies for more than one category, try to find out which aspect is the most important one in this response and use this aspect for categorizing the response.

The coding scheme has three levels of specificity. The code for a category indicates the level of specificity, the more digits a code has the more specific it is. For example, code 11 is a subcategory of code 1; code 111 is a subcategory of code 11. Always use the most specific category possible. Use a superordinate category if the response does not give more specific information to assign it to a subcategory or if something specific is said which does not match any of the subcategories.

Remember that each response was given as an association to a presented term. In the following, we call these terms “steps”.

**Supplementary Table 3.1**

Categories of the coding scheme

| **Code** | | | **Category** | **Description** | **Example(s)** |
| --- | --- | --- | --- | --- | --- |
| **Level 1** | **Level  2** | **Level 3** |  |  |  |
| 1 |  | | Requirements | The response indicates that the step will not work in isolation but requires some additional action.  No further specification is mentioned as to what is required. Or what is said does not match any of the subcategories 11 to 13. | - *Won’t work on its own.* |
|  | 11 |  | Requirement on international level | The response indicates that some action on an international or global level is required.  It is not specified what the international requirement is. Or what is said does not match subcategories 111 or 112. | - *Can’t be accomplished by one country alone.* |
|  |  | 111 | Need for international agreements | The response indicates that international agreements or some other cooperation across national borders is required. | - *Important to cooperate with the rest of the world in order to decrease pollution.* |
|  |  | 112 | Need for monitoring targets | The response indicates that international agreements or cooperation have to be monitored. | - *Important to have binding agreements that include sanctions if they are broken.* |
|  | 12 |  | Requirement on the level of national policies | The response indicates that some political action or policy on the national level is required.  It is not specified what kind of political action or policy is needed. Or what is said does not match subcategories 121 to 124. | - *Our politicians have to do more.* |
|  |  | 121 | Regulation via incentives | The response indicates that the government (nation) should introduce regulations that reward certain actions. | - *More people will choose to use an electric car when there is free parking for electric cars.* |
|  |  | 122 | Regulation via punishments | The response indicates that the government (nation) should introduce regulations that punish certain actions. | - *Airplane travel might be decreased by having higher taxes.* |
|  |  | 123 | Need for facilitation (available infrastructure) | The response indicates that structural changes are needed to promote behaviour change. | - *More people will use bicycles, if there are more bicycle lanes in the city.* |
|  |  | 124 | Need to increase knowledge (fund research) | The response indicates that more information is required, both in science and among the public. | - *We need more research in this field.* - *We need to inform the public.* |
|  | 13 |  | Requirement on the level of the citizens within a society | The response indicates that some change is required that concerns the citizens in a society.  It is not specified what kind of change is needed. Or what is said does not match subcategories 131 to 134. | - *People have to change.* |
|  |  | 131 | Need to change behaviour/lifestyles | The response indicates that there is a need for changes in behaviours and/or lifestyles. | - *People have to change their behaviour.* |
|  |  | 132 | Need to change attitudes/values | The response indicates that there is a need for changes in attitudes and/or values. | - *People have to change their attitudes.* |
|  |  | 133 | Need for collective action | The response indicates that there is a need for collective action. | - *Everybody needs to participate.* |
|  |  | 134 | Need to increase awareness | The response indicates that people need to become more involved about the issue. | - *We need increased awareness and political engagement concerning our own consumption*. |
| 2 |  | | Consequences | The response refers to potential consequences of the step. These can be positive or negative.  It is not specified what kind of consequence or who (or what) would be affected. Or what is said does not match subcategories 21 to 23. | - *That would have bad consequences.* - *That would have good consequences.* |
|  | 21 |  | Personal consequences | The response refers to consequences of the step that affect the respondent him-/herself or people in general. The consequences can be positive or negative.  The consequences are not further specified. Or what is said does not match subcategories 211 to 216. | - *That would have negative consequences for me.* - *That would be beneficial for me.* - *That would have disadvantages for people.* - *That would be good for people.* |
|  |  | 211 | Personal time resources | The response indicates that the step affects how much time people have available. | - *Buses take longer time than driving.* |
|  |  | 212 | Personal financial resources | The response indicates that the step affects how much financial resources people have at their disposal. | - *Trains are more expensive than flights.* - *Commuting by car is cheaper than public transport.* |
|  |  | 213 | Personal comfort | The response indicates that the step affects personal comfort. | - *The economy bulb gives less comfortable light.* - *Taking the train is convenient.* |
|  |  | 214 | Personal social interactions | The response indicates that social interactions are affected. | - *Carpooling is more social than driving alone.* |
|  |  | 215 | Personal health effects | The response indicates that personal health is affected. | - *It is healthy to walk more.* - *On public transport, you can catch the flu from other people.* |
|  |  | 216 | Personal freedom | The response indicates that personal freedom is impaired. | - *Reducing flights limits the traveling possibilities, what about my summer holiday?* |
|  | 22 |  | Societal consequences | The response refers to consequences of the step that affect society. The consequences can be positive or negative.  The kind of societal consequence is not further specified. Or what is said does not match subcategories 221 or 222. | - *That would affect our society.* |
|  |  | 221 | Social risks | The response indicates that the step poses a risk for society. | - *It deteriorates how people live together in communities.* |
|  |  | 222 | Social justice | The response indicates that the step has social implications. It can be fair, or it can lead to differences in opportunities/influence. | - *Affects the people with less money, but not the rich.* |
|  | 23 |  | Environmental consequences | The response refers to consequences of the step for the natural environment. The consequences can be positive or negative.  The kind of environmental consequence is not further specified. Or what is said does not match subcategories 231 to 233. | - *That would affect the environment.* |
|  |  | 231 | Environmental pollution | The response indicates that the step will affect the degree of pollution. | - *Driving together in a car rather than alone will reduce overall CO2 emissions.* |
|  |  | 232 | Environmental preservation | The response indicates that the step will contribute to preserving the environment. | - *Public transport uses less energy than driving your own car.* |
|  |  | 233 | Environmental aesthetics | The response indicates that the step has implications for how the environment looks. | - *Windmills are ugly and take up a lot of space.* |
| 3 |  | | Evaluation | The respondent expresses an evaluation, but does not clearly indicate to which aspect of the step he/she refers.  Or what is said does not match subcategories 31 to 36. | - *Remarkable.* - *Interesting.* |
|  | 31 |  | Evaluation concerning feasibility | The response indicates that the step is (or is not) feasible. | - *Can’t be accomplished.* - *That would be easy.* |
|  | 32 |  | Evaluation concerning effectiveness | The response indicates that the step is (or is not) effective. | - *This has little effect.* - *This is like a drop in the ocean.* |
|  | 33 |  | Evaluation concerning importance | The response indicates that the presented step is or is not important.  It is not specified in which sense the step is (or is not) important. Or what is said does not match subcategories 331 or 332. | - *That would matter a lot.* |
|  |  | 331 | Importance for the present | The response indicates that the step is important as of today. | - *It’s important that we do this today.* |
|  |  | 332 | Importance for the future | The response indicates that the step is important in the future. | - *This will be an important energy source in the future.* |
|  | 34 |  | Expression of skepticism | The respondent expresses general skepticism, without further specification. Or what is said does not match subcategories 341 or 342. | - *I don’t believe in it.* |
|  |  | 341 | Skepticism towards underlying intentions | The response implies suspiciousness and skepticism towards the reasons for implementing the step. | - *Expensive tickets are just to earn money, not to save the environment.* |
|  |  | 342 | Skepticism towards the scientific bases | The response implies suspiciousness and skepticism towards the scientific bases of the step. | - *Who is financing the research on this?* |
|  | 35 |  | Expression of affective valence | The respondent expresses an affective evaluation. It is not clear whether the evaluation is positive or negative. | - *I have no strong feelings about this.* |
|  |  | 351 | Positive affect | The respondent expresses positive affect. | - *Good.* - *Great.* |
|  |  | 352 | Negative affect | The respondent expresses negative affect. | - *Bad.* - *Scary and dangerous.* |
|  | 36 |  | Expression of conflicting aspects | The response indicates that the step has different aspects that conflict (or contradict each other).  The type of conflict is not specified. Or what is said does not match subcategories 361 to 362. | - *That has positive and negative sides.* |
|  |  | 361 | Conflict between different impacts | The respondent links both positive and negative factors with the step. | - *Tastes boring, but is good for the environment.* - *Good for the environment, but expensive.* |
|  |  | 362 | Conflict between different generations | The response implies differences in how one generation acts compared to another generation. | - *Future generations will have to pay the price for this.* |
| 4 |  | | Prevalence | The response refers to the prevalence of the step, indicating how widespread (or rare) the step is.  No further specification concerning the context of the prevalence is given. Or what is said does not match subcategories 41 or 42. | - *That’s everywhere.* - *That’s hardly to be seen.* |
|  | 41 |  | Prevalence with respect to personal actions | The respondent indicates that he/she is (or is not) engaging in the step.  It is not clear whether the response indicates high or low personal prevalence. Or what is said does not match subcategories 411 or 412. | - *I don’t know whether I am going to do this.* |
|  |  | 411 | Respondent is already doing it | The response indicates that the respondent is already partaking in the step. | - *I do this every day.* |
|  |  | 412 | Respondent lacks motivation | The response indicates that the respondent is not partaking in the step, because he/she lacks motivation. | - *I don’t bother doing this.* |
|  | 42 |  | Prevalence among certain social groups | The response indicates that the respondent believes the step is prevalent among certain social groups.  The social group is not specified. Or what is said does not match subcategories 421 or 422. | - *Some people do this.* |
|  |  | 421 | Prevalence among certain subcultures | The respondent links certain subcultures with the step. | - *That’s something done by hippies.* |
|  |  | 422 | Prevalence among demographic groups | The respondent links certain demographic groups with the step. | - *Younger people do this.* |
|  |  |  |  |  |  |
| **Remnant categories** | | | | | |
|  | 51 |  | Mere description | The respondent gives a mere description or rephrasing of the step. | - *Runs without fossil fuels (in response to “Electric cars”).* - *Produces energy from water (in response to “Hydropower”).* - *Turbines (in response to “Hydropower”).* - *Busses (in response to “Public transportation”)* - *Radioactive (in response to “Nuclear power”)* |
|  | 52 |  | Non-codeable response | The response does not match any of the categories. |  |
|  | 53 |  | Don’t know | The respondent indicates that he/she does not know an answer. | - *Don’t know.* - *Haven’t heard about it.* |
